# A Functional Kinase Is Necessary for Cyclin-Dependent Kinase G1 (CDKG1) to Maintain Fertility at High Ambient Temperature in *Arabidopsis*

**DOI:** 10.3389/fpls.2020.586870

**Published:** 2020-11-10

**Authors:** Candida Nibau, Despoina Dadarou, Nestoras Kargios, Areti Mallioura, Narcis Fernandez-Fuentes, Nicola Cavallari, John H. Doonan

**Affiliations:** ^1^Institute of Biological Environmental and Rural Sciences (IBERS), Aberystwyth University, Aberystwyth, United Kingdom; ^2^School of Life Sciences, University of Warwick, Coventry, United Kingdom; ^3^Institute of Science and Technology Austria, Klosterneuburg, Austria

**Keywords:** cyclin-dependent kinase, alternative splicing, fertility, pollen, *Arabidopsis*, temperature

## Abstract

Maintaining fertility in a fluctuating environment is key to the reproductive success of flowering plants. Meiosis and pollen formation are particularly sensitive to changes in growing conditions, especially temperature. We have previously identified cyclin-dependent kinase G1 (CDKG1) as a master regulator of temperature-dependent meiosis and this may involve the regulation of alternative splicing (AS), including of its own transcript. *CDKG1* mRNA can undergo several AS events, potentially producing two protein variants: CDKG1L and CDKG1S, differing in their N-terminal domain which may be involved in co-factor interaction. In leaves, both isoforms have distinct temperature-dependent functions on target mRNA processing, but their role in pollen development is unknown. In the present study, we characterize the role of CDKG1L and CDKG1S in maintaining *Arabidopsis* fertility. We show that the long (L) form is necessary and sufficient to rescue the fertility defects of the *cdkg1-1* mutant, while the short (S) form is unable to rescue fertility. On the other hand, an extra copy of CDKG1L reduces fertility. In addition, mutation of the ATP binding pocket of the kinase indicates that kinase activity is necessary for the function of CDKG1. Kinase mutants of CDKG1L and CDKG1S correctly localize to the cell nucleus and nucleus and cytoplasm, respectively, but are unable to rescue either the fertility or the splicing defects of the *cdkg1-1* mutant. Furthermore, we show that there is partial functional overlap between CDKG1 and its paralog CDKG2 that could in part be explained by overlapping gene expression.

## Introduction

Maintaining fertility under fluctuating environmental conditions is key to the reproductive success of flowering plants. Meiosis and pollen formation are particularly sensitive to environmental fluctuations, and mild stresses can result in major yield losses, for example, in cereals (Dolferus et al., 2011; Asseng et al., 2015; Kiss et al., 2017; Zhao et al., 2017; Lippmann et al., 2019; Alae-Carew et al., 2020). It is thus imperative to gain a better understanding of the mechanisms by which changing environmental variables are sensed and how these are translated into growth and differentiation programs. We have previously identified the *Arabidopsis* cyclin-dependent kinase G1 (CDKG1) protein as having an important role in maintaining pollen fertility and yield at high ambient temperature (Zheng et al., 2014).

Cyclin-dependent kinases are important regulators of many cellular processes ranging from cell division to cell death (Malumbres, 2014). By binding to their cognate cyclin in a tightly regulated manner, they phosphorylate target proteins, thus altering their activities. This leads to the activation or inactivation of signaling cascades that ultimately result in altered programs for growth and development. CDKs are present in all the organisms studied so far and their activities conserved across kingdoms (Dorée and Galas, 1994; Malumbres, 2014). In *Arabidopsis*, there are seven classes of CDK kinases named A–G with distinct but overlapping functions. CDKA and CDKB are thought to be the only bona fide cell cycle regulators, while CDKC, CDKD, and CDKE are proposed to regulate transcription, while CDKF is a CDK activator protein (Loyer et al., 2005; Menges et al., 2005; Doonan and Kitsios, 2009). Remarkably, the plant CDKG group is closely related to the kinases found in the Ph1 locus in wheat that are thought to be involved in maintaining homologous chromosome pairing in this polyploid species (Kitsios and Doonan, 2011; Greer et al., 2012). However, in plants, the role of CDKG in various cellular mechanisms is less well defined than the equivalent group CDKG10/11 in animals (Loyer et al., 2005).

In *Arabidopsis*, CDKG proteins are encoded by two closely related genes, *CDKG1* and *CDKG2*, interacting with the common co-factor/regulator Cyclin L1 (Van Leene et al., 2010). Plants lacking CDKG1 show normal vegetative growth and are sterile when grown at high ambient temperature but fertile at lower temperatures (Zheng et al., 2014). In contrast, plants lacking CDKG2 are fully fertile at both high and low temperatures (Zheng et al., 2014). Our recent work has shown that, at high ambient temperature, CDKG1 is required for meiotic chromosome pairing and recombination by helping to stabilize recombination intermediates (Zheng et al., 2014; Nibau et al., 2020b). At low temperature, chromosome pairing and recombination are not affected by the absence of CDKG1 (Zheng et al., 2014; Nibau et al., 2020b).

Splicing is the process by which intronic sequences are removed from the primary transcript to generate mature mRNA. The combinatorial use of alternative splice sites means that multiple transcripts can be produced from a single pre-mRNA in a process called alternative splicing (AS). The alternative transcripts may have different stabilities or may give rise to proteins with different activities. In addition, AS is a wide-spread and tightly regulated process that responds to developmental and environmental cues providing a rapid and flexible mechanism to amplify the complexity of the genome and to precisely modulate cell differentiation and development (Staiger and Brown, 2013; Lee and Rio, 2015). In higher plants, it is thought that 60–70% of genes can undergo AS (Reddy et al., 2013; Chamala et al., 2015; Zhang et al., 2017).

Both CDKG1 and CDKG2 have been shown to mediate the AS of downstream genes. In vegetative tissues, CDKG1 regulates the AS of the splicing factor *U2AF65A*, and in pollen, it regulates the AS of *CalS5* mRNA (Huang et al., 2013; Cavallari et al., 2018). CDKG2 regulates the AS of *FLOWERING LOCUS M* (*FLM*) to fine tune flowering time in *Arabidopsis* according to environmental temperature (Nibau et al., 2020a). Importantly, in vegetative tissues CDKG2 controls the temperature-dependent AS of CDKG1 (Cavallari et al., 2018). At first inspection, *CDKG1* appears to lack introns. Due to a suspected retrotransposition event in the *Arabidopsis* lineage (Zhang et al., 2005), the *CDKG1* mRNA does not need to be spliced to be translated into a full length protein. Despite this, conserved splicing signals and alternative introns exist both in the coding and in the UTR regions of *CDKG1* mRNA (Cavallari et al., 2018). If the intron is retained, a nuclear-localized full-length protein is produced, that we named CDKG1L. Removal of the first intron erases the first start codon of the mRNA that putatively produces a shorter isoform (CDKG1S), lacking two out of four SR domains for protein-protein interaction and the Nuclear Localization Signal (NLS). CDKG1S is localized to both the nuclear and cytoplasmic compartments (Cavallari et al., 2018). The ratio between the long (L) and short (S) forms is regulated by temperature and CDKG2. In the wild type, at low temperatures, mostly the L form is produced, while at higher temperatures there is production of both the L and the S forms and this is dependent on CDKG2 (Cavallari et al., 2018). Recent evidence reveals distinct functions for the L and S forms in the AS of *U2AF65A* in vegetative tissues. While CDKG1L was necessary to maintain normal AS of *U2AF65A* at low temperatures, at high temperatures, the presence of CDKG1S was necessary for the correct splicing of *U2AF65A* (Cavallari et al., 2018). A thermo-sensitive AS signaling cascade involving the CDKG kinases may, therefore, translate changes in ambient temperature into changes of the abundance of CDKG1L and CDKG1S isoforms and subsequent differential gene expression (Cavallari et al., 2018). Despite this, the functions of CDKG1L and CDKG1S during meiosis and pollen formation are still unknown. In the present work, we examine the function of CDKG1L and CDKG1S during pollen formation and their effect on plant fertility. By introducing different CDKG1 splice variants into the *cdkg1-1* mutant, we show that CDKG1L can rescue the mutant phenotype, restoring fertility, and wild type splicing patterns while CDKG1S cannot. In addition, we show that an active kinase is necessary for CDKG1 to maintain pollen formation and fertility. Finally, we show that there is partial functional redundancy between the two members of the CDKG kinase family, CDKG1 and CDKG2.

## Materials and Methods

### Construct Generation and Plant Transformation

*CDKG1SC-GFP*, *CDKG1L-GFP*, *CDKG1S-GFP*, and *CDKG2-GFP* sequences described in Cavallari et al. (2018) were cloned into pDONR207 by PCR. The *CDKG1* (At5g63370) and *CDKG2* (At1g67580) promoters were cloned into the pDONRP4-P1R vector. All the primers used for cloning are listed in Supplementary Table 1. The different promoter and coding sequence combinations were cloned into the pB7m24GW,3 (Karimi et al., 2002) destination vector by multisite Gateway™ cloning and transformed into *Agrobacterium tumefaciens* strain GV3101 by electroporation. *Arabidopsis cdkg1-1* and Columbia (Col-0) plants were transformed by floral dip (Clough and Bent, 1998). Single insertion, BASTA-resistant plants were selected and insertion was confirmed by PCR and the presence of GFP.

### Protein Modeling and Kinase Mutant Generation

The structural model of CDKG1 kinase (UNIPROT, Uniprot Consortium, 2015, identification code Q9FGW5) was derived by homology modeling using M4T (Fernandez-Fuentes et al., 2007a,b) using the structures of CDK12 (Dixon-Clarke et al., 2015; PDB code: 4un0) and CDK13 (Greifenberg et al., 2016; PDB code: 5efq) obtained from Protein Databank (Berman et al., 2000). The sequence identity between CDKG1 sequence and both structures used as templates is above 40%, hence ensuring structural similarity (Baker and Sali, 2001). The quality and stereochemistry of the model was assessed using Prosa-II (Sippl, 1993) and PROCHECK, respectively (Laskowski et al., 1993).

The ATP binding pocket was modeled and the Aspartic acid (D) residue known to be important for activity in other related kinases (Hemerly et al., 1995) was identified at position 426 in CDKG1L. This conserved Aspartic acid residue (D) was mutated to the positively charged Asparagine (N) for the CDKG1SC, CDKG1L, and CDKG1S constructs described above by site-direct mutagenesis using the Gene-Art Site-Directed Mutagenesis kit (Invitrogen) following manufacturer’s instructions. The primers used for the mutagenesis are listed in Supplementary Table 1. Mutated sequences were confirmed by sequencing and used for plant transformation as described above.

### Plant Growth

The wild type Columbia (Col-0) and the previously characterized *cdkg1-1* T-DNA insertion mutant line (SALK_075762, Zheng et al., 2014) that does not produce full length RNA were obtained from the Nottingham *Arabidopsis* Stock Centre. Seeds were sown in a mixture of Levington F2 compost and sand (80:20%, respectively) and grown in growth chambers under long-day conditions (16 h light/8 h dark, the light intensity was 150 *μ*molm−2s−1, provided by Sylvania 840 lamps) with the temperature set at 23°C.

### RNA Extraction and qPCR

Total RNA was extracted from young inflorescences (no open flowers) from the first 2–3 inflorescence stems using the RNeasy Plant Mini kit with in-column DNAse treatment following the manufacturer’s protocol (Qiagen). One microgram total mRNA was used to generate cDNA using the SuperScript® III First-strand Synthesis kit (Invitrogen). For RT-PCR, 50 ng of cDNA was used per reaction. The *PP2AA3* (AT1G13320) was used as a reference (Czechowski et al., 2005). Quantitative polymerase chain reaction (qPCRs) were performed using the LightCycler 480 (Roche). Typically, 10 ng of cDNA were used in a 20 μl reaction containing 0.25 μM of each primer and 10 μl LightCycler® 480 SYBR Green I Master (Roche). Each reaction was done in triplicate, GFP primers were used to detect expression of the transgene and *PP2AA3* (AT1G13320) was used as a reference (Czechowski et al., 2005; see Supplementary Table 1 for primer sequences). Data were analyzed using the LightCycler® 480 Software (Roche). The data shown are averages ± SE of at least three experiments.

### Splicing Assays

For the splicing assays, inflorescence cDNA for each line was generated as described above. Typically, 50 ng of cDNA was used in a PCR reaction with primers specific for the detection of the presence of intron 8 (labeled as intron 6 in the original publication) in *CalS5* as described in Huang et al. (2013) and for the splicing variants of *ATU2AF65A* (Cavallari et al., 2018; Supplementary Table 1). The amplification products were run on a 2% gel and examined for the presence of the spliced and unspliced variants.

### Pollen Viability Staining

To determine pollen viability, flowers that had just opened were collected into 3:1 Ethanol: Acetic acid fixative and stored at room temperature for at least 2 days. For whole anthers imaging, anthers were placed in a drop of Alexander’s stain on a microscope slide and gently squashed with a coverslip before sealing with nail varnish. Anthers were allowed to stain for 48 h before imaging. Representative images are shown. For pollen viability counts, anthers were placed in Alexander’s stain, cut open with a razor blade, and gently tapped to release pollen. After 24 h staining, viable (purple) and non-viable (blue) pollen were counted. At least 100 pollen grains from six anthers from three different plants were scored (Supplementary Table 2).

### Fertility Counts

For fertility seed counts, the main inflorescence stem was harvested when the first siliques started to become yellow. The first five siliques were discarded and the number of seeds in the following 10 siliques was counted under a dissecting microscope. At least 30 siliques from three different plants were counted per transformed line and two to three different transformants per construct. Results shown represent mean ± interquartile range. Statistical significance was calculated using ANOVA, with *post hoc* pairwise *t*-tests using non-pooled SD and Bonferroni correction.

### Microscopy

For root imaging, seeds were sown on vertical plates containing 0.5xMS media (salts and vitamins) pH = 5.6 and 1% agar. Plates were kept in the cold for 2 days to synchronize germination and moved to a growth chamber set at 23°C continuous light for 4 days. After this time, seedlings were mounted in microscope slides and imaged using a Leica TCS SPE confocal laser scanning microscope (CLSM) controlled by Leica LAS-AF software. Images are single confocal sections. Multiple plants per line and multiple lines were observed and representative images are shown.

## Results

### The L but Not the S Form of CDKG1 Can Rescue the *cdkg1-1* Mutant Fertility Phenotype

**Figure 1:**
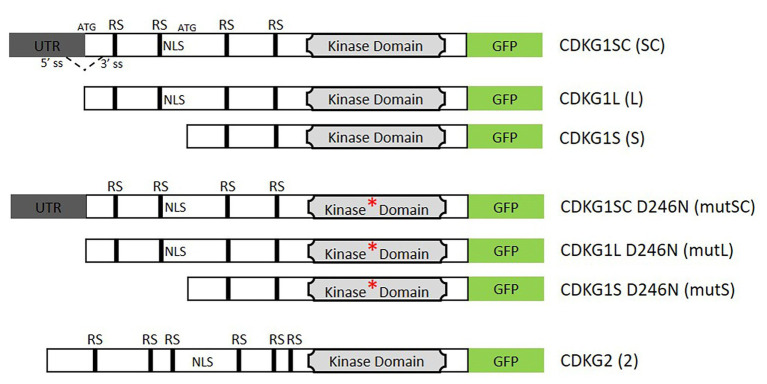
Schematic representation of the constructs used in this study. The coding region is represented by the open box and the 5'UTR in gray. The GFP coding sequence in shown green. The kinase domain is depicted by a light gray box and the asterisk indicates the D426N mutation of the kinase active site. Arginine/Serine rich motifs (RS) and indicated by black boxes, (NLS, nuclear localization signal). The splicing donor (5'SS) and acceptor sites (3'SS) and the two possible start codons (ATG) are indicated in the splice competent (SC) form. The dashed line indicates the potential splicing event. In the absence of splicing, the first ATG is used and the long (L) form produced, if splicing occurs then only the second ATG site will be present and only the short (S) form can be produced. The represented cassettes were cloned under the control of the cyclin-dependent kinase G1 (CDKG1) promoter unless otherwise stated and transformed into a *cdkg1-1* or Col-0 background.

**Figure 2:**
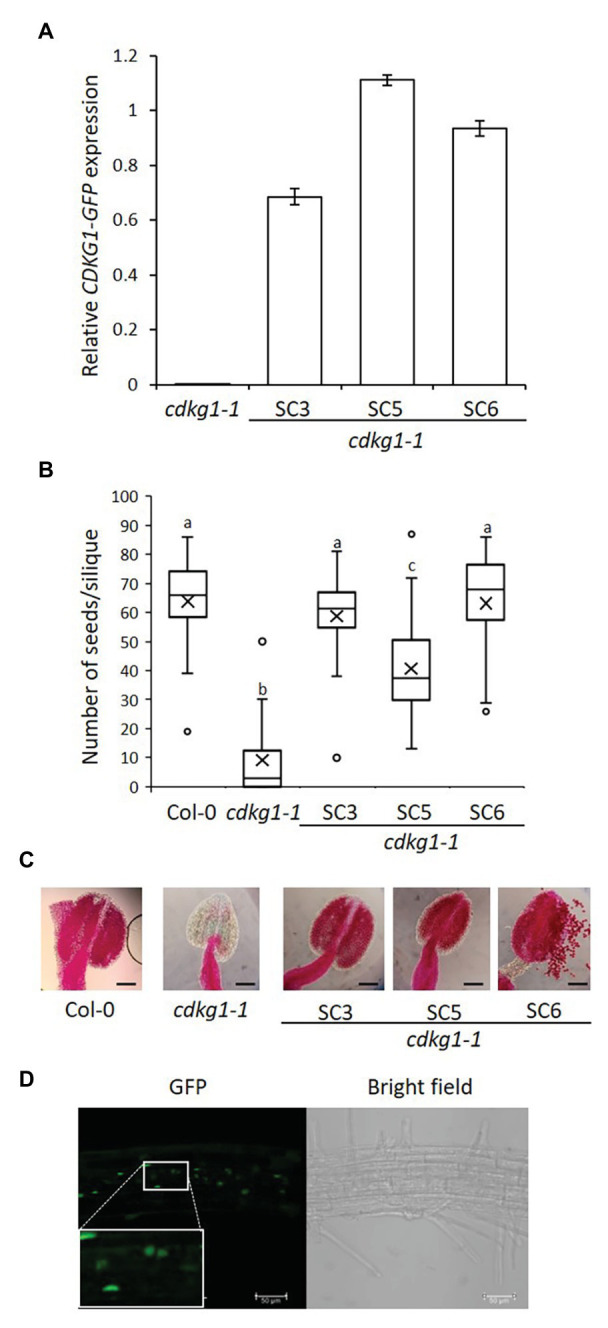
The SC form of CDKG1 can rescue the *cdkg1-1* mutant phenotype. **(A)** Expression levels of the *CDKG1SC-GFP* transcript as detected by qPCR in young inflorescences. Three independent lines expressing CDKG1SC-GFP in a *cdkg1-1* background were tested. The *cdkg1-1* mutant was used as a control. **(B)** Fertility counts of the transformed plants. The average number of seeds per silique was determined for Col-0, *cdkg1-1*, and three independent lines expressing CDKG1SC-GFP under the control of the CDKG1 promoter in a *cdkg1-1* mutant background. Data represent average and the interquartile range for 30 siliques from three plants. Superscript letters represent significance groups for *p* < 0.001. **(C)** Pollen viability staining of the lines above. Round carmine-colored pollen grains are viable, while white/blue shrunken pollen is not viable. Scale bar = 100 *μ*m. **(D)** The majority of the CDKG1SC-GFP protein is found in the nucleus of root cells in plants grown at 23°C and a small proportion observed in the cytoplasm. Images are single confocal sections. The GFP channel (green) and a bright field image are shown. Inset shows a magnification of the selected area. Scale bar = 50 μm.

**Figure 3:**
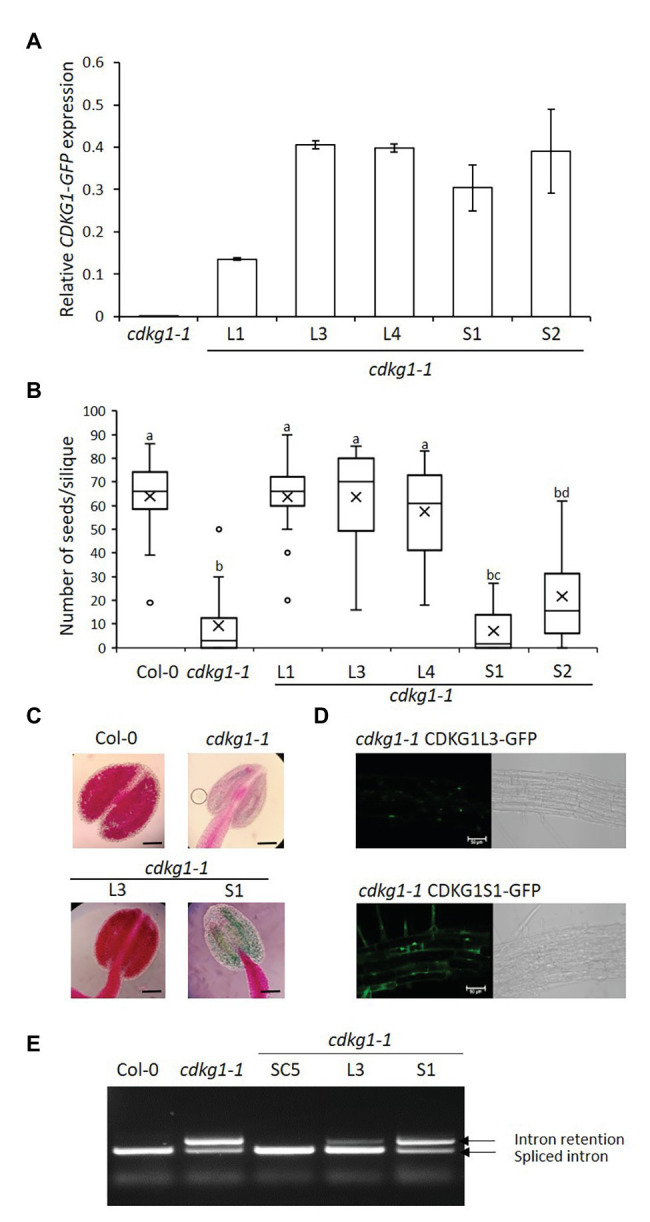
The L but not the S form of CDKG1 can rescue the *cdkg1-1* mutant phenotype. **(A)** Expression levels of the *CDKG1L-GFP* and *CDKG1S-GFP* transcripts as detected by qPCR in young inflorescences. Three independent lines expressing CDKG1L-GFP and two lines expressing CDKG1S-GFP in a *cdkg1-1* background were tested. The *cdkg1-1* mutant was used as a control. **(B)** Fertility counts of the transformed plants. The average number of seeds per silique was determined for Col-0, *cdkg1-1*, and lines expressing CDKG1L-GFP and CDKG1S-GFP under the control of the CDKG1 promoter in a *cdkg1-1* mutant background. Data represent average and the interquartile range for 30 siliques from three plants. Superscript letters represent significance groups for *p* < 0.001. **(C)** Pollen viability staining of the lines above. Round carmine-colored pollen grains are viable, while white/blue shrunken pollen is not viable. Scale bar = 100 μm. **(D)** The CDKG1L-GFP protein localizes to the nucleus of root cells, while the CDKG1S-GFP protein can be found both in the nucleus and in the cytoplasm of root cells in plants grown at 23°C. Images are single confocal sections acquired using the same settings. The GFP channel (green) and a bright field image are shown. Scale bar = 50 μm. **(E)** Splicing profile of the CalS5 mRNA in young inflorescences of the different mutants. In the wild type Col-0 only the fully spliced form is observed, while in the *cdkg1-1* mutant, both spliced and intron retention forms are observed.

We have previously shown that, in somatic tissues, the different isoforms of CDKG1 have a differential temperature-dependent effect on splicing of the target gene, *U2AF65A* (Cavallari et al., 2018). In order to determine the function of the different isoforms in maintaining plant fertility at higher ambient temperatures, we cloned *CDKG1* splice competent (SC) form (capable of producing both the L form and the S form Cavallari et al., 2018), the L form and the S form, all under the control of the endogenous *CDKG1* promoter (*pCDKG1*; Figure 1). We then introduced the constructs into the *cdkg1-1* mutant (Zheng et al., 2014), isolated single insertion homozygous lines, and confirmed transgene expression by qPCR (Figures 2A, 3A). As the *cdkg1-1* mutant only displays the fertility defects at high ambient temperature, all the experiments were performed at 23°C.

As previously described (Zheng et al., 2014), the *cdkg1-1* mutant has dramatically reduced fertility when grown at 23°C but this defect is fully reversed by the introduction of the *pCDKG1::CDKG1SC-GFP* construct (Figure 2B). The reduced pollen viability of the *cdkg1-1* mutant was also rescued as seen by Alexander’s stain (Figure 2C; Supplementary Table 2). In wild type Col-0, anthers are filled with round, carmine-colored viable pollen, while anthers of the *cdkg1-1* mutant are shrunken and virtually empty (Figure 2C). In the lines expressing *CDKG1SC-GFP*, the anthers are full of viable pollen, comparable to the wild type (Figure 2C). As previously shown (Cavallari et al., 2018), at 23°C the majority of the CDKG1SC-GFP protein is found in the cell nucleus with only faint labeling seen in the cytoplasm (Figure 2D).

Similarly to the SC form, expression of the nuclear-localized CDKG1L form (Figures 3A,D) is also able to rescue the fertility and pollen viability phenotype of the *cdkg1-1* mutant (Figures 3B,C). In contrast, *cdkg1-1* mutant plants transformed with the S form, although expressing the construct to levels similar to the L form (Figure 3A) were infertile and unviable pollen was found in their anthers (Figures 3B,C; Supplementary Table 2). As shown previously, the CDKG1S-GFP protein was found to localize both to the nucleus and cytoplasm (Figure 3D; Cavallari et al., 2018).

Another consequence of the *cdkg1-1* mutation is the aberrant splicing of the *CalS5* gene (Huang et al., 2013). In the wild type Col-0 young inflorescences, only the fully spliced version of the *CalS5* gene is detected but, in the *cdkg1-1* mutant, an aberrant transcript containing the fully retained intron 8 (referred to as intron 6 in the original publication) is also present (Figure 3E). As seen with the fertility phenotype, the SC form of CDKG1 fully rescues the splicing defect. In plants carrying the L form, although some unspliced transcript is still observed, the majority of the transcripts do not contain intron 8 (Figure 3E). The splicing pattern of the S form is comparable to the *cdkg1-1* mutant alone (Figure 3E), again showing that the S form alone is unable to rescue the *cdkg1-1* mutant. The inability of CDKG1S to rescue the *cdkg1-1* mutant phenotype was also observed when we looked at the splicing of the *ATU2AF65A* mRNA in young inflorescences. In this case, three major AS transcripts are observed, mRNA1, mRNA2, and mRNA3 (Cavallari et al., 2018). The splicing index (SI) for *ATU2AF65A* was calculated as the ratio of mRNA2/(mRNA2 + mRNA3). In wild type Col-0, mRNA2 is more abundant than mRNA3 and the SI is higher; the opposite is observed in the cdkg1-1 mutant where mRNA3 becomes more abundant and this has a lower SI (Supplementary Figure 1; Cavallari et al., 2018). The SC and L forms can fully rescue the splicing defect of the *cdkg1-1* mutant by increasing the SI, while the S form cannot (Supplementary Figure 1). This observation is different from what we observed in leaves where the S form could rescue the splicing defect of *ATU2AF65A* at high temperatures (Cavallari et al., 2018), suggesting tissue-specific functions for CDKG1 isoforms.

### Increased Expression of CDKG1L Decreases Fertility

**Figure 4:**
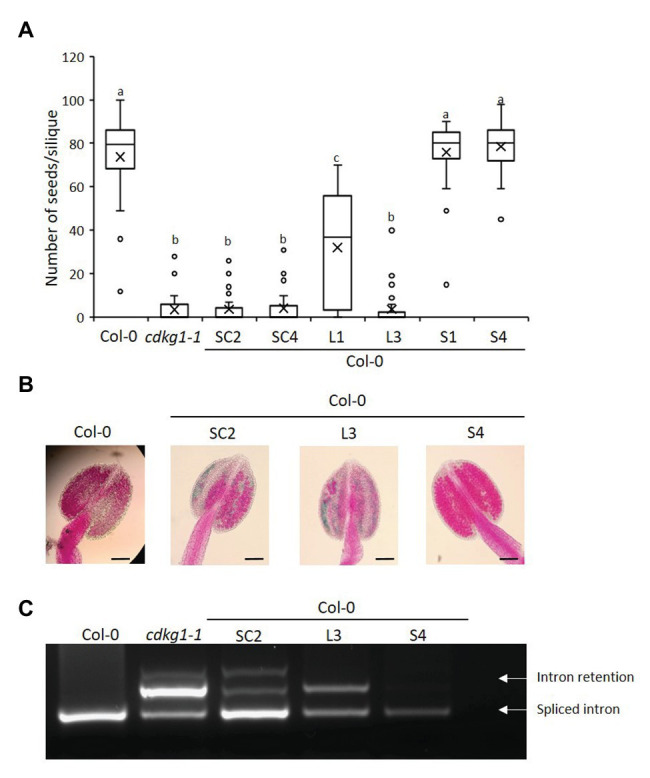
Increased expression of CDLG1L decreases fertility in a Col-0 background. **(A)** Fertility counts of Col-0 plants transformed with the different CDKG1-GFP constructs. The average number of seeds per silique was determined for Col-0, *cdkg1-1*, and lines expressing the various constructs under the control of the CDKG1 promoter in a Col-0 background as indicated. Data represent average and the interquartile range for 30 siliques from three plants. Superscript letters represent significance groups for *p* < 0.001. **(B)** Pollen viability staining of the lines above. Round carmine-colored pollen grains are viable, while white/blue shrunken pollen is not viable. Scale bar = 100 μm. **(C)** Splicing profile of the CalS5 mRNA in young inflorescences of the different lines as described above. The CDKG1SC-GFP and CDKG1L-GFP lines show the intron retention observed in the *cdkg1-1* mutant. The extra high molecular weight band observed in some of the samples is possibly a result of RNA heteroduplex formation.

To test the effect of increased expression of the different CDKG1 isoforms on the fertility of *Arabidopsis* plants, we introduced the constructs described above into the wild type Col-0 background. We found all the constructs to be expressed (Supplementary Figure 2). When we looked at the fertility of homozygous T3 plants, we found that plants expressing either the SC or the L form had reduced seed set when compared to the wild type Col-0 (Figure 4A). While the anthers of the wild type Col-0 were full of carmine-colored, round, viable pollen grains, the anthers of plants expressing SC or L contained dramatically reduced amounts of pollen and some of it was blue-stained, shrunken, unviable pollen (Figure 4B; Supplementary Table 2). In contrast, increased expression of the S form had no effect on fertility or on pollen amount and viability (Figures 4A,B). The negative effect of over expressing the CDKG1SC and L forms was also seen when we looked at *CalS5* splicing (Figure 4C). In Col-0 plants expressing the S form, only the fully spliced *CalS5* transcript is observed, but, in SC and L-expressing Col-0 plants, the intron retention form is also observed, just like in the *cdkg1-1* mutant (Figure 4C). This suggests that the increased expression of CDKG1L mRNA or protein has a negative effect on the endogenous CDKG1 signaling pathways. Indeed, the levels of endogenous *CDKG1* transcripts are generally reduced in plants with an extra copy of *CDKG1SC* and *CDKG1L*, while the extra copy of *CDKG1S* has no effect on the endogenous levels of the *CDKG1* transcript (Supplementary Figure 2B).

To test whether the effect of an extra copy is due to gene silencing or requires CDKG kinase activity, we introduced mutated forms of CDKG1where the ATP binding pocket had been mutated. These presumptive kinase-dead versions have no effect on endogenous transcript levels (Supplementary Figure 2C), suggesting that increased levels of CDKGL, but not CDKGS, kinase impacts negatively on fertility.

### Promoter Swap Experiments Reveal Overlapping Functions Between the CDKG1 and CDKG2 Proteins

**Figure 5:**
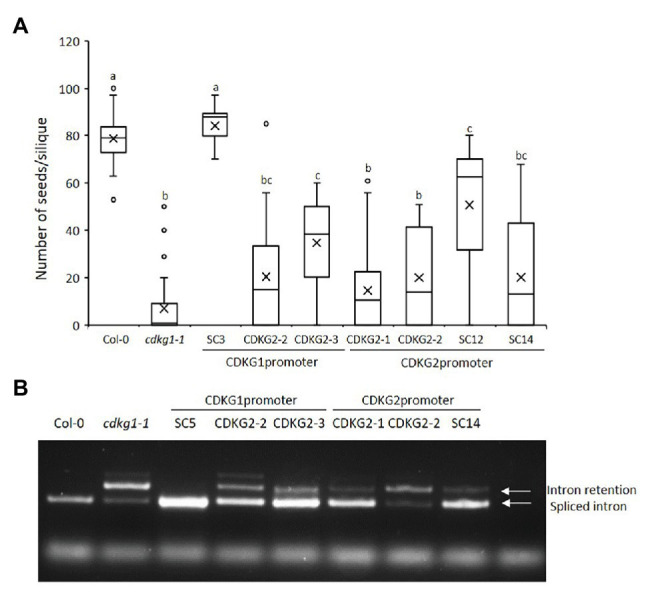
CDKG2 is able to partially rescue the *cdkg1-1* phenotype. **(A)** Fertility counts of the transformed plants. The average number of seeds per silique was determined for Col-0, *cdkg1-1*, and lines expressing CDKG1 and CDKG2 under the control of the CDKG1 promoter or the CDKG2 promoter in a *cdkg1-1* mutant background as indicated. Data represent average and the interquartile range for 30 siliques from three plants. Superscript letters represent significance groups for *p* < 0.001. **(B)** Splicing profile of the CalS5 mRNA in young inflorescences of the different lines as described above. In the wild type Col-0 only the spliced form is observed, while in the *cdkg1-1* mutant both spliced and intron retention are observed. The extra high molecular weight band observed in some of the samples is possibly a result of RNA heteroduplex formation.

Studies in somatic tissues suggest that both CDKG1 and its homolog CDKG2 perform distinct functions in regulating gene splicing (Huang et al., 2013; Cavallari et al., 2018; Nibau et al., 2020a). On the other hand, while each single mutant is viable, we cannot recover a double *cdkg1-1cdkg2-1* mutant, suggesting that their functions do overlap and are essential. Indeed, based on publicly available data, the expression of *CDKG1* (At5g63370) and *CDKG2* (At1g67580) overlap in most tissues and treatment conditions (Supplementary Figure S3; Schmid et al., 2005; http://bar.utoronto.ca/eplant/). To test if CDKG2 could replace CDKG1 in maintaining fertility at high ambient temperature, we introduced *CDKG2* back into the *cdkg1-1* mutant either under its own promoter or the *CDKG1* promoter. We confirmed *CDKG2* expression by the two different promoters (Supplementary Figure 4A). When expressed from the *CDKG1* promoter, CDKG2 could partly rescue the *cdkg1-1* mutant phenotype. When *CDKG2* was expressed from its own promoter, it also partially rescued the fertility phenotype (Figure 5A). A similar effect was seen in the pollen viability assays (Supplementary Figure 4B; Supplementary Table 2). In addition, *CDKG1* was also able to partially rescue the *cdkg1-1* phenotype when expressed from the *CDKG2* promoter. The fertility results were mirrored by what was seen when we looked at the splicing of *CalS5*. The CDKG2 protein was able to only partially rescue the splicing defect when expressed from the *CDKG1* or its own promoter (Figure 5B). Furthermore, the CDKG1 protein expressed from its own promoter was more effective at reversing the splicing defect in *cdkg1-1* than when expressed from the *CDKG2* promoter (Figure 5B). This suggests that there is only partial expression and functional overlap between CDKG1 and CDKG2.

### An Intact Kinase Domain Is Necessary for the Function of CDKG1

**Figure 6:**
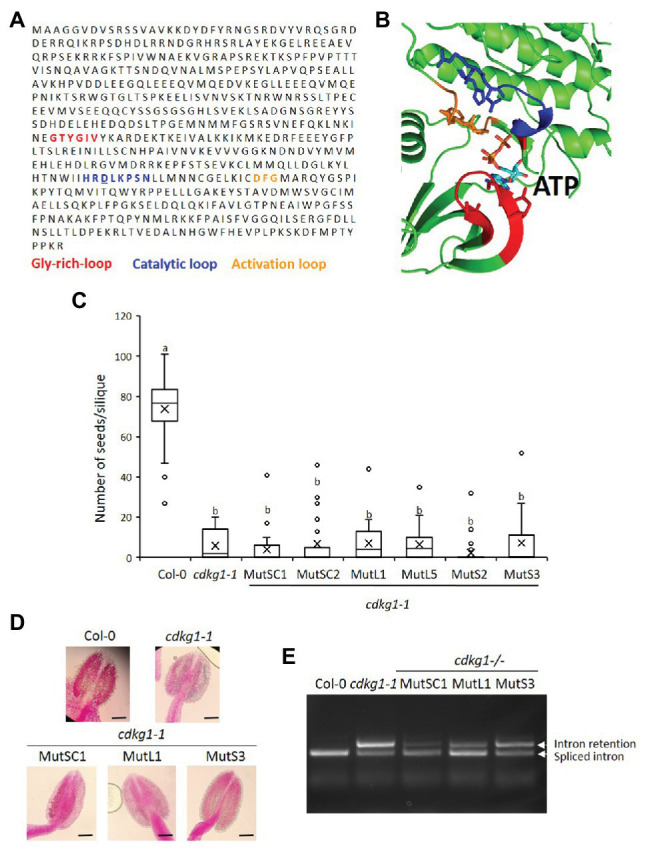
An active kinase is necessary for the function of CDKG1. **(A)** Sequence of CDKG1 protein. Highlighted are the regions important for kinase activity as indicated. The Aspartic acid **(D)** residue mutated to Asparagine (N) is underlined. **(B)** Cartoon representation of the structural model of the CDKG1 active site bound ATP. The Glycine-rich loop is highlighted in red, the catalytic loop blue, the activation loop orange, and ATP light blue. The structure of CDKG1 was rendered using PyMol (http://pymol.org). **(C)** Fertility counts of the plants transformed with the kinase mutant constructs. The average number of seeds per silique was determined for Col-0, *cdkg1-1* and lines expressing the various constructs under the control of the CDKG1 promoter in a *cdkg1-1* mutant background as indicated. Data represent average and the interquartile range for 30 siliques from three plants. Superscript letters represent significance groups for *p* < 0.001. **(D)** Pollen viability staining of the lines above. Round carmine-colored pollen grains are viable, while white/blue shrunken pollen is not viable. Scale bar = 100 μm. **(E)** Splicing profile of the CalS5 mRNA in young inflorescences of the different lines. In the wild type Col-0 only the spliced form is observed, while in the *cdkg1-1* mutant both spliced and intron retention are observed. None of the kinase mutant variants is able to rescue the *cdkg1-1* mutant splicing defect.

Cyclin-dependent kinases physically interact with their cognate cyclin(s), together bind to specific substrates and phosphorylate them to alter their activity, stability, or other attributes. The kinase domain of CDKG1 has a widely conserved ATP-binding pocket necessary for its activity. In order to determine if the activity of CDKG1 is necessary for its function maintaining fertility at high ambient temperature, we modeled the CDKG1 ATP binding pocket based on other available CDK crystal structures: CDK12 and CDK13. The structure of CDKG1 bound to ATP was derived by homology modeling, for which CDK12 (Dixon-Clarke et al., 2015) and CDK13 (Greifenberg et al., 2016) shared the highest sequence identity to CDKG1, above 40%, therefore justifying their use as templates. It is important to stress that CDKG1 presents all the functional elements present in all kinase belonging to this fold: the glycine-rich, the activation, and the catalytic loop (Figures 6A,B). The charged residues within the ATP binding pocked are conserved across kingdoms (Figures 6A,B), including the Aspartic acid (D) at position 426 in CDKG1 that when mutated abolishes activity in other CDKs (Hemerly et al., 1995). We changed the conserved negatively charged Aspartic acid (D) into the positively charged Asparagine (N) in the CDKG1SC, CDKG1L, and CDKG1S constructs by site directed mutagenesis (Figure 1) and introduced them into *cdkg1-1* mutant plants. We detected expression of the different mutated *CDKG1* constructs (Supplementary Figure 5) and the mutant CDKG1-GFP proteins show the same subcellular localization as the one observed for the non-mutated versions (Supplementary Figure 6; Cavallari et al., 2018). Despite this, none of the mutated CDKG1-GFP proteins was able to rescue the *cdkg1-1* mutant phenotype in terms of fertility (Figure 6C), pollen viability (Figure 6D; Supplementary Table 2), or *CalS5* splicing (Figure 6E).

**Figure 7:**
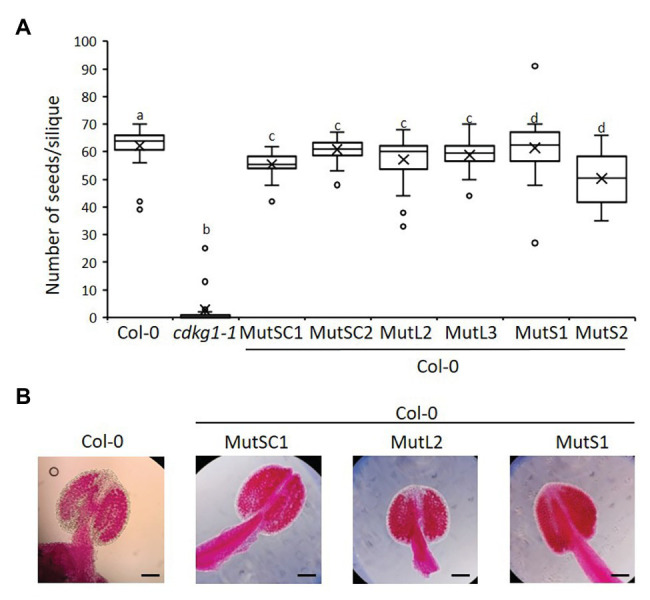
The mutated kinase forms of CDKG1 do not act was dominant negative kinases in a Col-0 background. **(A)** Fertility counts of the kinase mutant transformed plants. The average number of seeds per silique was determined for Col-0, *cdkg1-1*, and lines expressing the various constructs under the control of the CDKG1 promoter in a Col-0 background as indicated. Data represent average and the interquartile range for 30 siliques from three plants. Superscript letters represent significance groups for *p* < 0.001. **(B)** Pollen viability staining of the lines above. Round carmine-colored pollen grains are viable, while white/blue shrunken pollen is not viable. Scale bar = 100 μm.

The non-mutated CDKG1L had a negative effect on fertility when expressed at higher levels (Figure 4). To test if this was dependent on an active kinase, or if the kinase-dead mutants can act as a dominant negative by competing for endogenous substrates or cyclins, we introduced the different CDKG1 mutant forms in a Col-0 background. We observed no changes in their subcellular localization (Supplementary Figure 6), and the expression of the different forms (Supplementary Figure 5B) had no effect on plant fertility or the pollen viability of the Col-0 plants (Figures 7A,B). This indicates that the detrimental effect on plant fertility caused by increased expression of CDKG1L is dependent on the presence of an intact ATP-binding domain.

## Discussion

### CDKG1L and CDKG1S Perform Different Functions During Development

Alternative splicing of pre-mRNA has emerged not only as an important means of regulating mRNA stability but also as a way to diversify mRNA and protein functions. It is believed that AS is one of the main sources of protein diversity in eukaryotes (Nilsen and Graveley, 2010; Baralle and Giudice, 2017). In addition, while some events are constitutive, some are regulated at the tissue and/or at the developmental level and in response to environmental conditions (Nilsen and Graveley, 2010). We have previously shown that the *CDKG1* pre-mRNA can be alternatively spliced to produce two main isoforms that result in two different proteins. We have called the long form CDKG1L and a shorter version lacking the extended N terminal domain CDKG1S. The ratio between the two isoforms is modulated by temperature through CDKG2 (Cavallari et al., 2018). We previously reported that the ratio between the two isoforms in vegetative tissues is important to maintain function along the temperature range but, in this paper, we report that, in reproductive tissues, only the L form is necessary to maintain fertility. Thus, CDKG1L and CDKG1S have different functions in different tissues or during different developmental stages.

The observation that different protein isoforms can perform different functions is not unusual. It is suggested that 95% of humans and over 70% of *Arabidopsis* multi-exon genes undergo AS (Pan et al., 2008; Filichkin et al., 2010; Lu et al., 2010; Szakonyi and Duque, 2018). Even considering that a large proportion of the resulting transcripts contain premature stop codons or are degraded by the nonsense-mediated decay pathway (Severing et al., 2011), there is enormous potential to create proteins with different functions, and this might have provided an evolutionary advantage to the organisms. Indeed, several protein isoforms resulting from alternative splicing events have been shown to have distinct functions. Some examples include transcription factors, kinases, proteases, and cation channels (Oberwinkler et al., 2005; Subramanya et al., 2006; Végran et al., 2006; Eksi et al., 2013; Ganassi et al., 2014; Wang et al., 2019). Crucially, a mammalian homolog of CDKG, CDK11, is also present in two isoforms resulting from alternative transcript processing: CDK11^p110^ has functions in transcription and splicing, while CDK11^p58^ is specifically active during mitosis (Xiang et al., 1994; Loyer et al., 2008). A third isoform, CDK11^p46^ is produced by protease cleavage and is involved in Apoptosis (Beyaert et al., 1997). Interestingly, the rice OsRAD1 protein involved in DSBs repair during meiosis is present in three different isoforms resulting from AS events that alter the structure of the C-terminus domain. Despite all being expressed in anthers undergoing meiosis, only one of the isoforms (OsRAD1-1) was necessary and sufficient to rescue the meiotic defects of the *osrad1* mutant (Yuan et al., 2020).

While the S form does not seem to be necessary to maintain fertility, the corresponding mRNA is still produced in floral tissue and thus might perform other functions or modulate the stability of the *CDKG1L* transcript under certain conditions. The presence of the *CDKG1S* transcript might provide a feedback mechanism to maintain expression homeostasis by keeping a tight balance between the *CDKG1S* and *CDKG1L* mRNA forms. Another level of regulation can occur, whereby AS generates both productive and aberrant transcripts, and these compete to regulate the pool of available pre-mRNA, limiting the expression of active transcripts. Alterations in these processes can lead to imbalances in AS transcripts, resulting in meiotic defects and diseases like cancer (Kalsotra and Cooper, 2011; Marcel et al., 2011; Yap and Makeyev, 2016).

At the protein level, the distinct functions of CDKG1L and CDKG1S in different tissues could be due to the presence of different amounts of each protein or to the presence or absence of important co-factors. Another possibility is that the presence of the inactive S form interferes with the stability of CDKG1L complexes, thus regulating the number and/or activity of the protein complexes at a given time as it is the case with human receptor-like protein tyrosine phosphatases (Petrone and Sap, 2000; Xu and Weiss, 2002) and DNA methyltransferases (Gordon et al., 2013). Further experiments are needed to clarify the function of CDKG1S.

### CDKG1 and CDKG2 Have Partially Overlapping Functions

The suggestion that the *CDKG1* gene has arisen from a retrotransposition event from the *CDKG2* gene in the *Arabidopsis* lineage after the split from other Brassicaceae (Zhang et al., 2005; Beilstein et al., 2010) has raised questions related to their function and why both genes have been retained. Even though CDKG1 and CDKG2 proteins share 64% amino acid similarity, we and others have shown that the two kinases perform different functions (Huang et al., 2013; Zheng et al., 2014; Ma et al., 2015; Cavallari et al., 2018; Chen et al., 2018; Nibau et al., 2020a,b). On the other hand, we were not able to recover double *cdkg1-1cdkg2-1* mutants despite both single mutants showing only mild growth phenotypes. This suggests that their functions at least partially overlap. We knew from previous studies that expression of *CDKG2* was not able to rescue the splicing defects of the *cdkg1-1* mutant in somatic tissues so we asked the same question in reproductive tissues. CDKG2 can only partially rescue the fertility and splicing phenotype of the *cdkg1-1* mutant in reproductive tissues, suggesting that there is limited functional redundancy in these tissues. This general failure to complement might be explained by differences in the protein interaction networks for the two proteins, which share only a few common interacting partners and their expression is correlated with largely non-overlapping groups of genes (Supplementary Figure S7; Obayashi et al., 2009; http://atted.jp).

The expression patterns of both genes are similar through development and under different environmental conditions (Supplementary Figure S3; http://bar.utoronto.ca/eplant/). These observations and the demonstrated functional differences suggest that, despite similar expression patterns and similarity at the protein level, both CDKG1 and CDKG2 perform largely distinct functions in *Arabidopsis*. These distinct functions may be explained by the variation observed in the N terminal domain of both proteins, which is thought to influence the choice of interaction partners and by the different pattern of expression of co-factors and target proteins. Alternatively, fine spatio-temporal control of kinase activity may be required for optimal function as an extra copy of CDKG1 in Col-0 also leads to reduced fertility.

### An Active CDKG1 Kinase Is Necessary to Maintain Fertility in *Arabidopsis*

Cyclin-dependent kinases act by transferring a phosphate group from ATP to target molecules, and thereby altering the activity or conformation of the target proteins. The kinase domain, and specifically the ATP pocket, is conserved in CDKs across all kingdoms. Mutations in conserved residues in the ATP pocket are known to abolish kinase activity and this is also the case for CDKG1. CDKG1 proteins where the Aspartic acid at position 426 within the ATP pocket was mutated were unable to rescue the *cdkg1-1* fertility defect, indicating that the functions of CDKG1 during meiosis and pollen formation are dependent on its kinase activity. During male meiosis, CDKG1 is necessary to stabilize early recombination intermediates that are needed to maintain synapsis and normal levels of Class I crossovers (Nibau et al., 2020b). Based on the data obtained using versions with a mutated ATP binding pocket, we speculate that CDKG1 is doing this by phosphorylating meiotic proteins involved in early meiotic recombination. There is precedent for this from other systems: in *Caenorhabditis elegans*, CDK1 is known to phosphorylate the axis protein SYP1 and promote the formation of chromosome subdomains insuring correct chromosome segregation (Sato-Carlton et al., 2018). In mammal spermatogenesis, CDK2 activity is not only necessary to promote chromosome synapsis *via* its function at telomeres but also has been shown to localize to late recombination nodules and be necessary for class I CO formation (Palmer et al., 2019). In both cases, loss of CDK activity results in sterility. In *Arabidopsis*, weak loss-of-function alleles of the CDKA;1 kinase result in severe meiotic defects and the plants being completely sterile (Dissmeyer et al., 2007, 2009; Wijnker et al., 2019). Similar observations have been made using mutants in the cognate cyclins, cyclins CYC1A1;2 (also known as TARDY ASYNCHRONOUS MEIOSIS, TAM) and CYCB3;1 or SOLO DANCERS (SDS; Magnard et al., 2001; Azumi et al., 2002; Bulankova et al., 2013). Recent studies have identified the SC axis protein ASY1 as a target for phosphorylation by CDKA;1 (Yang et al., 2020), directly connecting CDK activity with chromosome pairing and CO formation. It is thus possible that the CDKG1 protein is performing a similar function by phosphorylating meiotic proteins recruiting them to the sites of recombination during early prophase.

An alternative, perhaps complementary, way in which CDKG1 kinase may be acting is by the regulation of the splicing of transcripts important for meiosis and pollen development. Indeed, CDKG1 is necessary for the correct splicing of the *CalS5* gene, crucial for pollen coat formation (Huang et al., 2013). Although not much is known in plants, during mammalian male germ line cell differentiation, there is a global transcript reprograming underpinned by changes on AS patterns (Naro et al., 2017). These changes are not only important to change the activity of protein networks during germ cell formation but also to guarantee that there is timely expression of specific transcripts (Naro et al., 2017). In budding yeast, mutation in the meiosis-specific *MER2* gene leads to meiotic recombination defects. Interestingly, although the *MER2* gene is expressed in mitosis and meiosis, it is only efficiently spliced to generate a functional gene product in meiotic cells (Engebrecht et al., 1991; Munding et al., 2010). More recently, MER2 has been identified as part of a regulatory splicing network including other meiotic-specific proteins that, together with transcriptional networks, ensure coherent gene expression during the meiotic programs (Munding et al., 2010).

It is thus reasonable to accept that meiosis-specific AS is a widespread phenomena across phylogeny and that similar processes may be at work in plants. In this scenario, CDKG1 could be part of the machinery regulating splicing at the early stages of meiosis. Many of the genes involved in early meiosis have annotated splice variants and CDKs have been shown to phosphorylate spliceosome components in a cell-cycle dependent manner (Murthy et al., 2018; Ryu et al., 2019). Specifically, CDK11, a CDKG homologue, phosphorylates components of the spliceosome and regulates pre-mRNA splicing (Hu et al., 2003).

### Increasing Copy Number of CDKG1 Reduces Fertility in Wild Type *Arabidopsis*

While CDKG1 is necessary to maintain fertility at high ambient temperature in *Arabidopsis*, adding an additional copy of the wild type CDKG1 has a detrimental effect on pollen formation and fertility. Plants expressing the functional CDKG1L form from the endogenous promoter in a wild type Col-0 background have significantly reduced fertility. This effect is probably due to the presence of an active kinase as the kinase dead form of CDKG1 does not have the same effect when introduced back to Col-0. In addition, an extra copy of *CDKG1S*, which is not able to rescue the fertility defect in the *cdkg1-1* mutant, does not have an effect on the fertility of Col-0 plants. The way the presence of an extra copy of *CDKG1* affects fertility is at least in part due to the effect on the endogenous transcript as the levels of endogenous *CDKG1* transcript are reduced in these plants, causing a phenotype similar to the *cdkg1-1* mutant. Observations that increasing or decreasing the activity of specific cell cycle regulators proteins produce similar phenotypic defects are often seen in cancer models, and there are some examples in plants as well (Samuel and Ellis, 2002; Feng, 2012; Wang et al., 2016), highlighting the tight regulatory systems these signaling molecules are under in the cellular environment.

Taken together, our results show that the role of CDKG1 in maintaining fertility at high ambient temperature is dependent on the presence of a full N-terminal domain and a functional ATP binding pocket. Furthermore, decreased or increased levels of CDKG, both, produce a mutant phenotype, suggesting that modulation of kinase activity is critical for optimum fertility, and that there are auto-feedback mechanisms yet to be characterized that regulate its expression. We suggest that CDKG1 might be acting through the phosphorylation of proteins important for the early stages of meiotic division and/or by regulating the splicing of the transcripts that produce these proteins.

## Data Availability Statement

The original contributions presented in the study are included in the article/Supplementary Material, further inquiries can be directed to the corresponding authors.

## Author Contributions

CN, NC, and JD conceived the project, designed research, and wrote the paper. CN, DD, NK, AM, and NF-F performed research. JD supervised the project and obtained funding. All authors contributed to the article and approved the submitted version.

### Conflict of Interest

The authors declare that the research was conducted in the absence of any commercial or financial relationships that could be construed as a potential conflict of interest.
