# Supplementary material for: A Functional Kinase Is Necessary for Cyclin-Dependent Kinase G1 (CDKG1) to Maintain Fertility at High Ambient Temperature in Arabidopsis
Source: Front Plant Sci. 2020 Nov 10;11:586870. doi: 10.3389/fpls.2020.586870 (PMC7683410; doi:10.3389/fpls.2020.586870)
Supplement: Supplementary file 1 [file Data_Sheet_1.pdf]

SUPPLEMENTARY MATERIAL

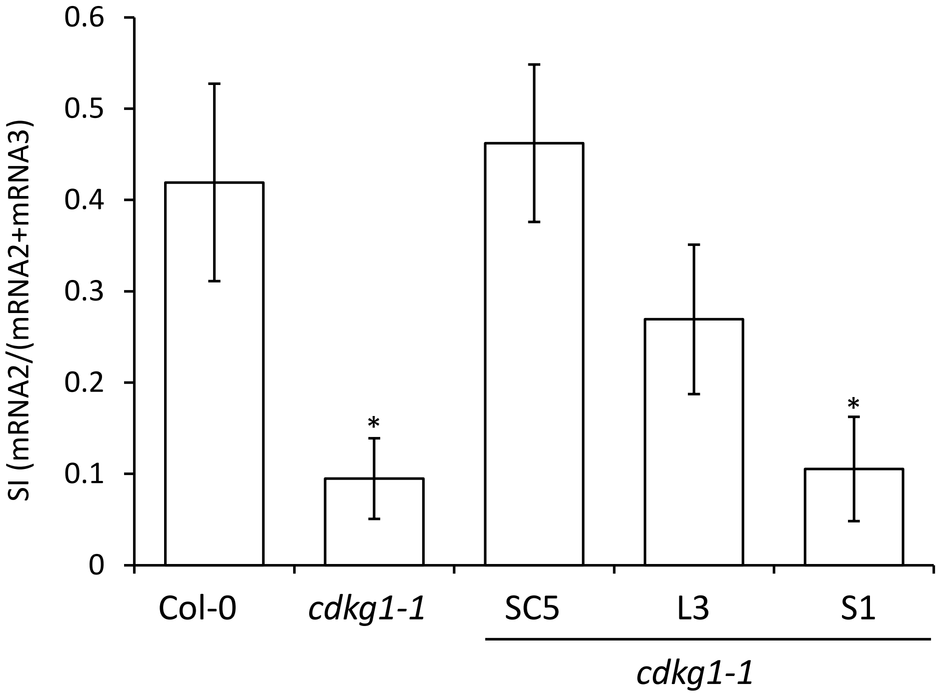

Supplementary Figure 1. CDKG1SC and CDKG1L are able to rescue the splicing defect of *U2AF65A* in flower buds of the *cdkg1-1* mutant but CDKG1S is not. The graph shows the splicing index (SI) of *U2AF65A* calculated as the ratio mRNA2/(mRNA2+mRNA3). In Col-0, mRNA2 is more abundant than mRNA3 so the SI is higher while the opposite is true for the *cdkg1-1* mutant. Both the CDKG1SC and CDKG1L can rescue the splicing defect of *cdkg1-1* but CDKGS can not. * indicates significantly different from the Col-0 for p<0.05.

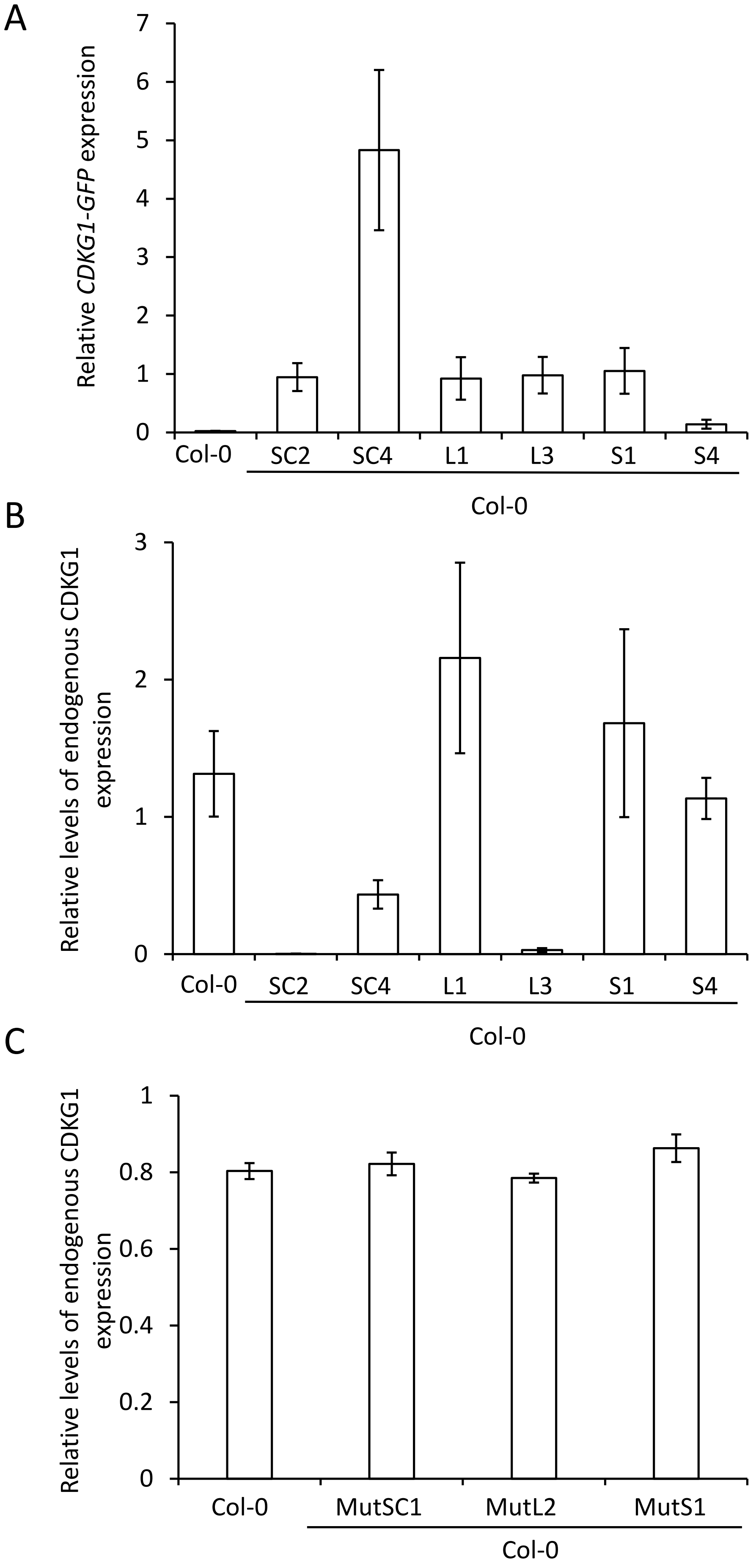

Supplementary Figure 2. Increased expression of CDKG1 isoforms in a Col-0 background.
(A) Expression levels of the different CDKG1-GFP transcript isoforms expressed in a Col-0 background as indicated, detected by qPCR of the GFP transcript. Col-0 was used as a control. Bars indicate average ±SE. **(B, C)** Expression of endogenous CDKG transcripts in Col-0 plants transformed with the intact **(B)** or mutated kinase constructs **(C)** as indicated, detected by qPCR. Expression of *PP2A* was used as a reference.

A

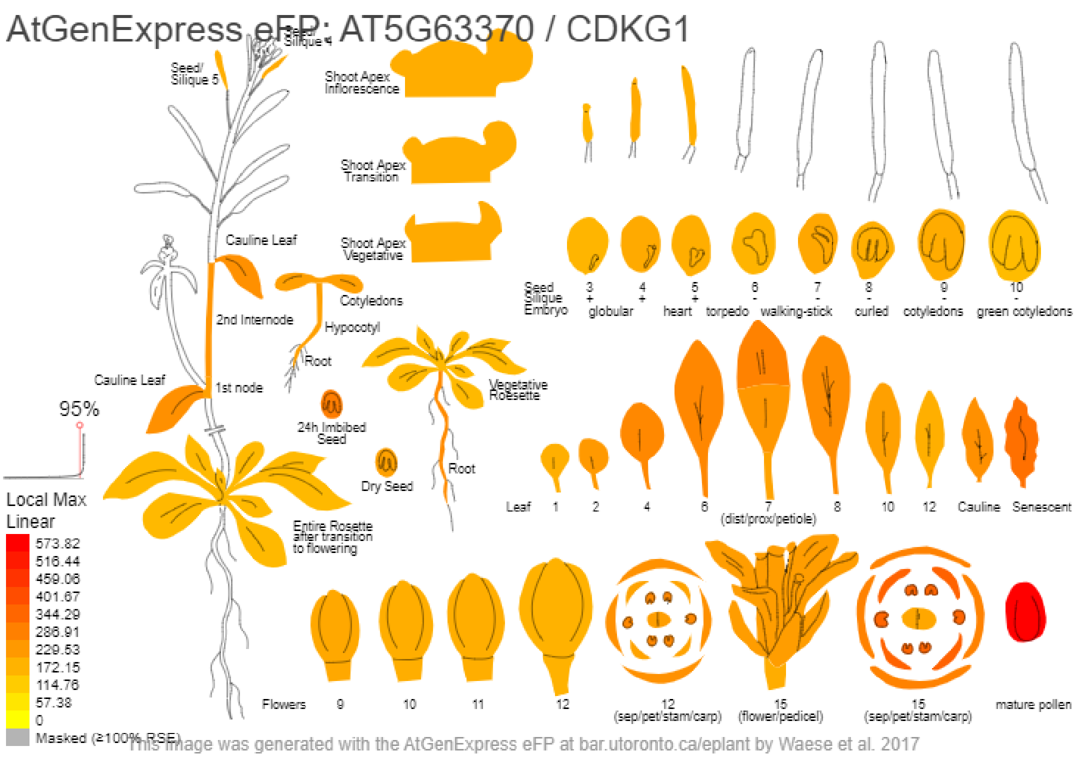

B

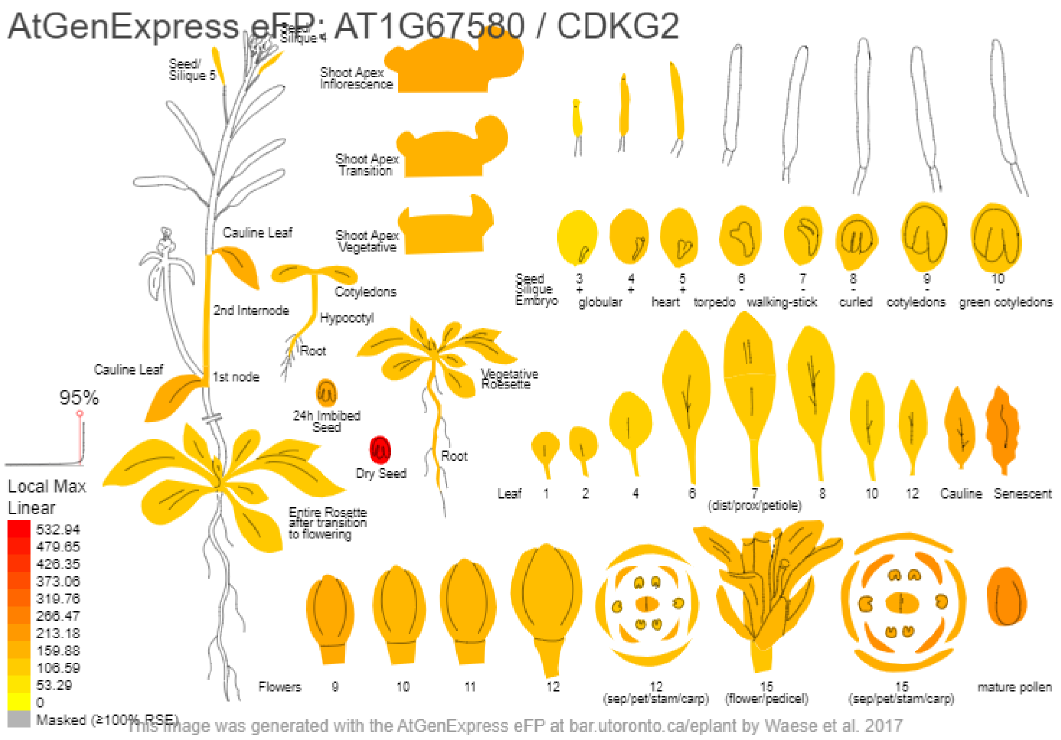

Supplementary Figure 3. Developmental expression profile for CDKG1 **(A)** and CDKG2 **(B)**. A heat map of expression is overlaid on the different plant developmental stages and organs.
Data are derived from Gene Expression Map of Arabidopsis Development: Schmid et al., 2005, and the Nambara lab for the imbibed and dry seed stages. Data are normalized by the GCOS method, TGT value of 100. Most tissues were sampled in triplicate. Based on the original eFP Browser by B. Vinegar, drawn by J. Alls and N. Provart. Vectorized image redrawn and programmed by J. Waese.

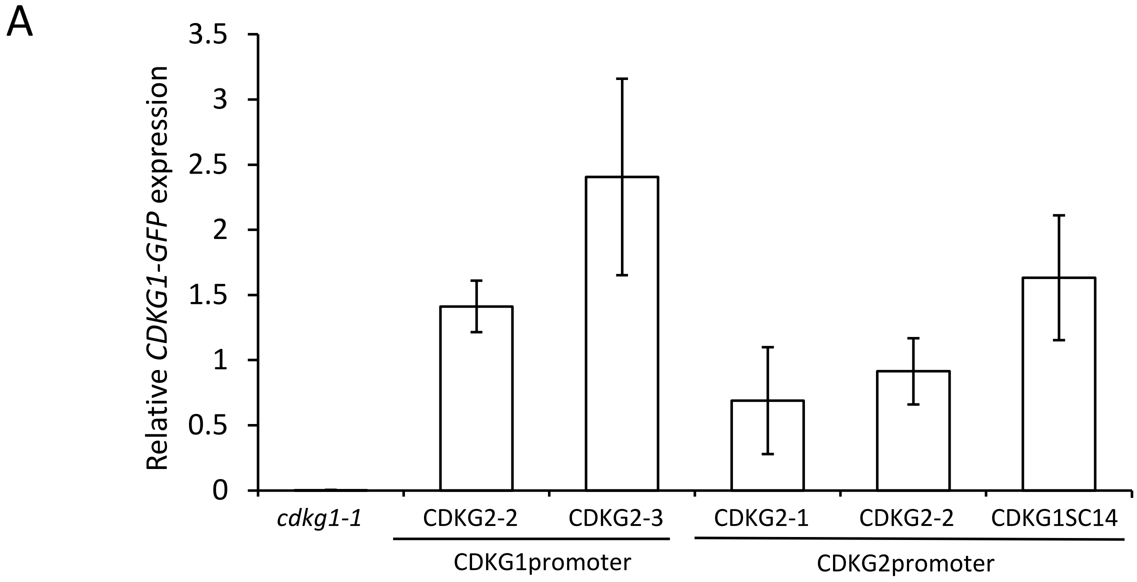

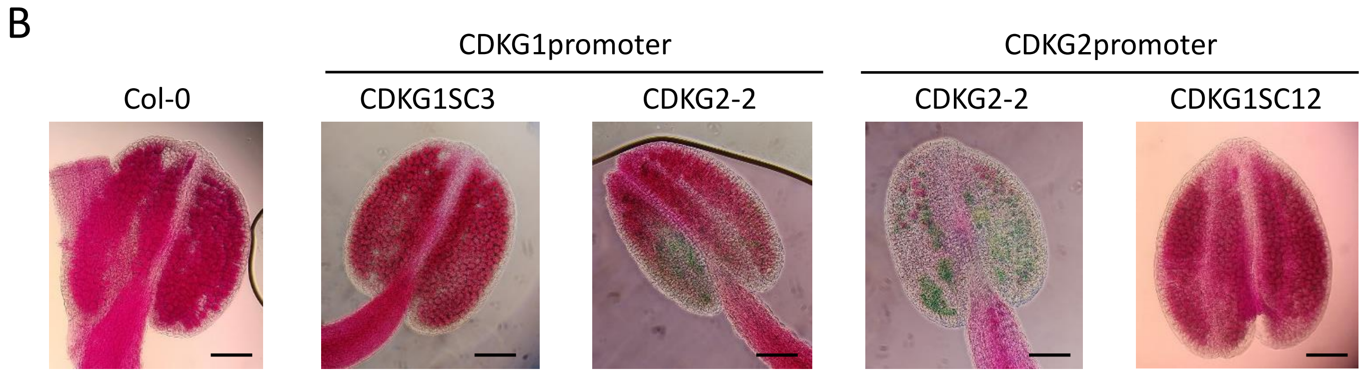

Supplementary Figure 4. CDKG2 is able to partially rescue the *cdkg1-1* phenotype.
(A) Expression levels of CDKG1 and CDKG2 transcripts expressed under the CDKG1 or CDKG2 promoter in the *cdkg1-1* background as indicated, detected by qPCR. The *cdkg1-1* mutant was used as a control. **(B)** Pollen viability staining of some of the lines above. Round carmine-coloured pollen grains are viable while white/blue shrunken pollen is not viable. Scale bar 100µm.

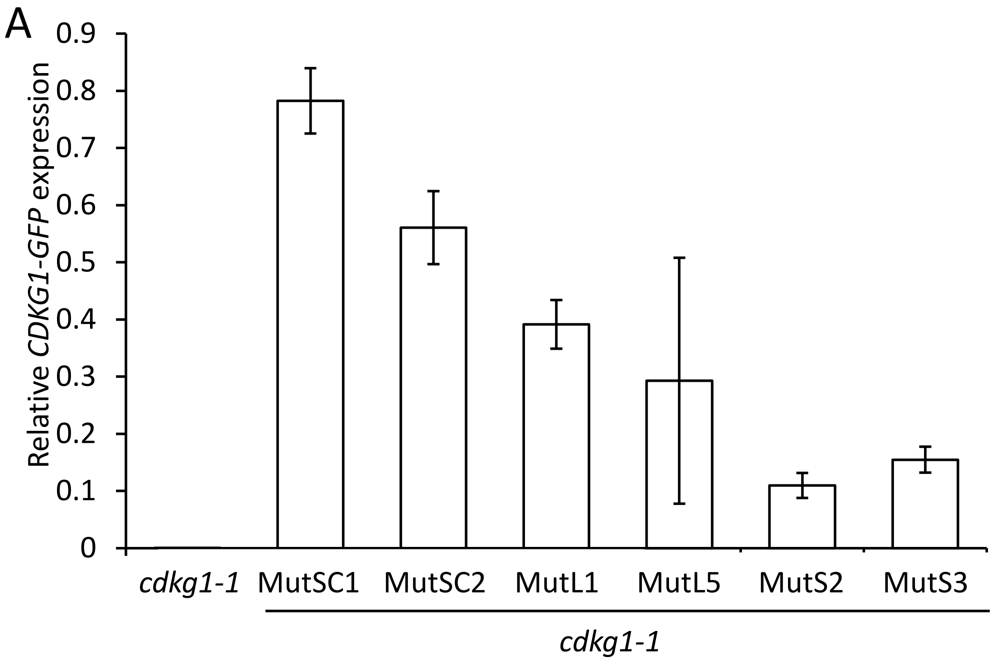

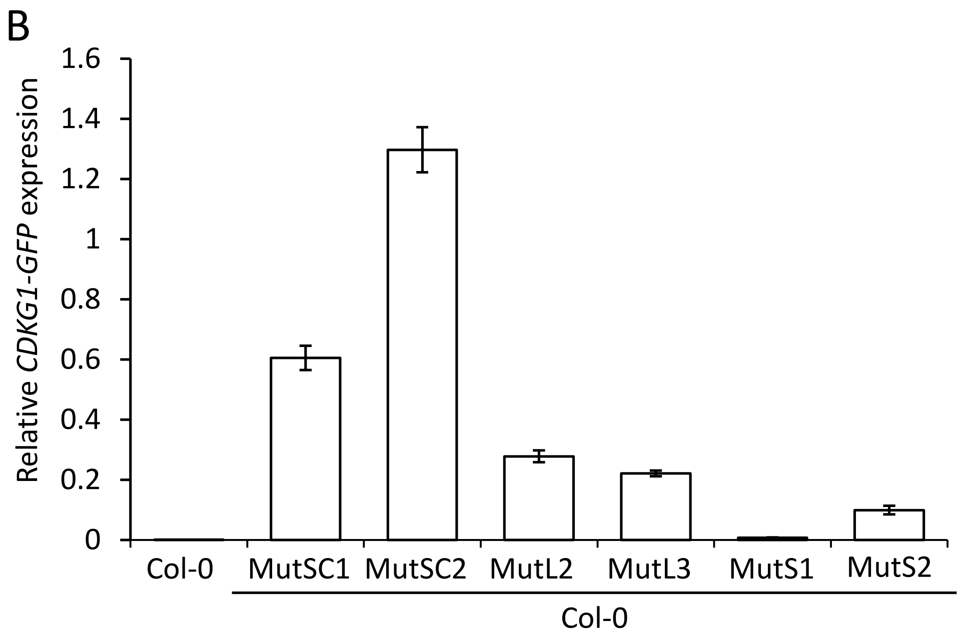

Supplementary Figure 5. Expression levels of the CDKG1-GFP mutant kinase transcripts in the *cdkg1-1* **(A)** and Col-0 **(B)** background as indicated, detected by qPCR. Two independent lines for each mutant construct in a *cdkg1-1* background were tested.

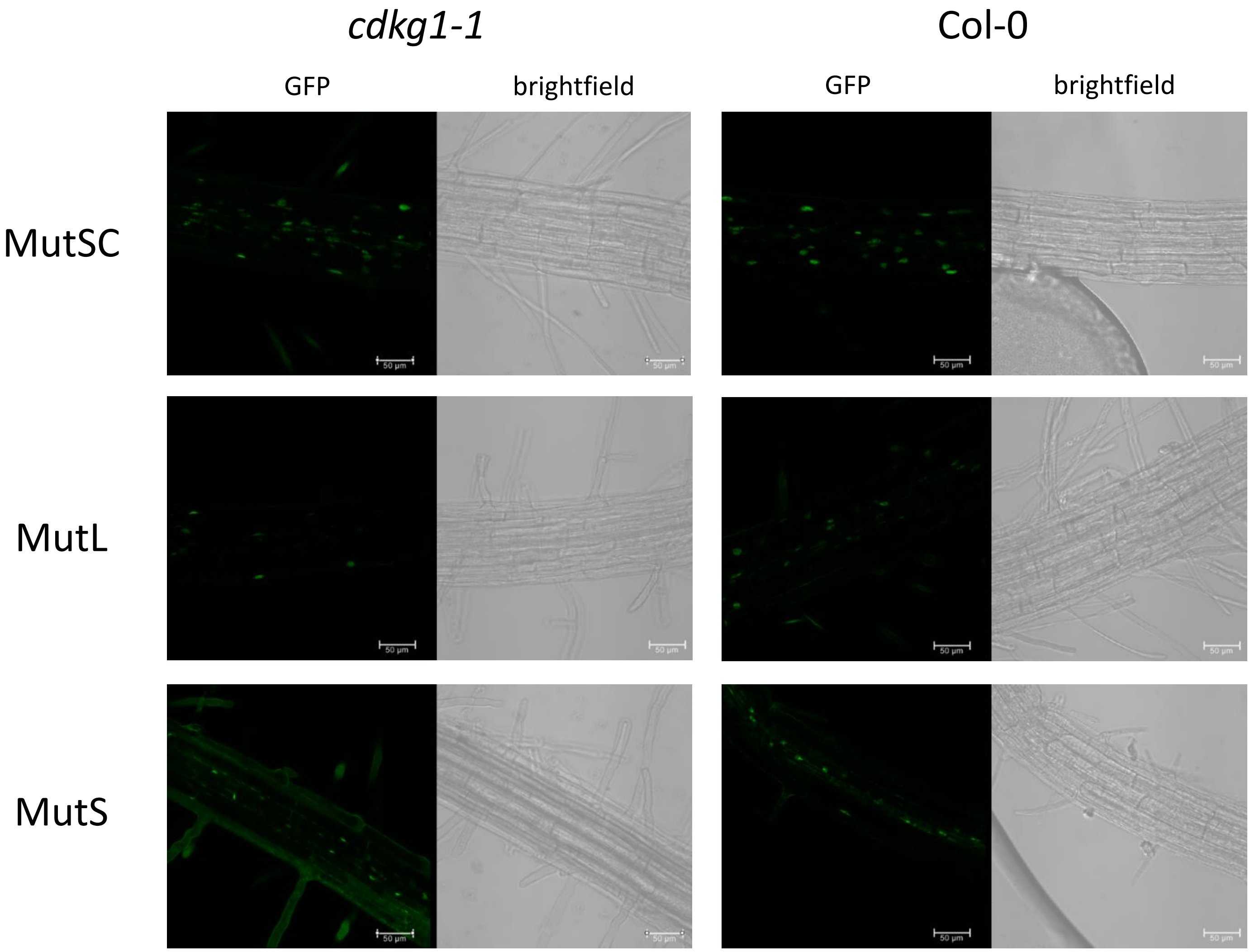

Supplementary Figure 6. The mutated kinase forms of CDKG1 show the same localisation pattern as the wild type forms in both the *cdkg1-1* mutant and the wild type Col-0 when grown at 23°C. The L form is exclusively nuclear localised and the S form is found both in the nucleus and cytoplasm. Images are single confocal sections all acquired under the same settings. The GFP channel (green) and a bright field image are shown. Scale bar=50µm.

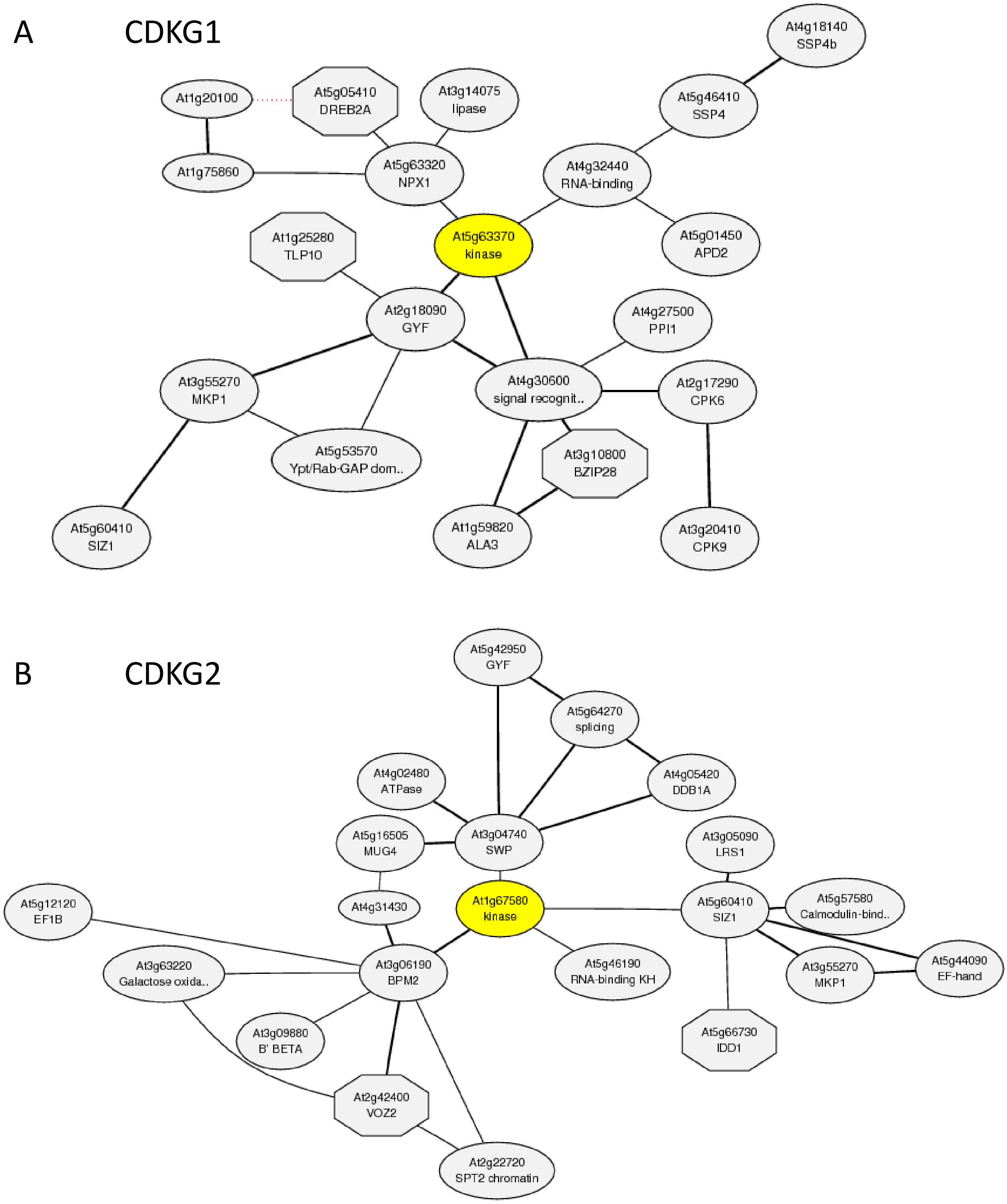

Supplementary Figure 7. Co-expression networks for CDKG1 (A) and CDKG2 (B) as generated by the ATED-II database (http://atted.jp, Obayashi et al. 2009). The CDKG kinases are indicated by a yellow shaded box, Solid lines indicate gene co-expression, octagon-shaped nodes indicate TF genes, and circular nodes indicate other types of genes. Red dashed lines indicate known protein-protein interactions.
