# Supplementary material for: A Functional Kinase Is Necessary for Cyclin-Dependent Kinase G1 (CDKG1) to Maintain Fertility at High Ambient Temperature in Arabidopsis
Source: Front Plant Sci. 2020 Nov 10;11:586870. doi: 10.3389/fpls.2020.586870 (PMC7683410; doi:10.3389/fpls.2020.586870)
Supplement: Supplementary file 2 [file Data_Sheet_2.pdf]

Supplementary Table 1: Primers used in this study.

Primer name	Primer Sequence	Purpose
CDKG1SC attB1	GGGGACAAGTTTGTACAAAAAAGCAGGCTTGCGC GTC TCC GAG AGG AAA ATT TAG	cloning
CDKG1 no stop attB2	GGGGACCACTTTGTACAAGAAAGCTGGGT TCCGCTTTGGAGGATATGTCG	cloning
CDKG1L attB1	GGGGACAAGTTTGTACAAAAAAGCAGGCTCGATGGCAGCAGGGGGTGTTGATG	cloning
CDKG1S attB1	GGGGACAAGTTTGTACAAAAAAGCAGGCTTGATGTCGCCAGAACCTAGTTATC	cloning
CDKG1p attB4	GGGGACAACTTTGTATAGAAAAGTTGGTCGTATCTCTGAGAATATGG	cloning
CDKG1p attB1R	GGGGACTGCTTTTTTGTACAAACTTGTCA ATT TTG TCA AGT GGC TCT A	cloning
CDKG2 attB1	GGGGACAAGTTTGTACAAAAAAGCAGGCTTGATGGCGGCTGGGAGGAATATAAG	cloning
CDKG2 no stop attB2	GGGGACCACTTTGTACAAGAAAGCTGGGTTGCCAAACAGACCGCCAGAG	cloning
CDKG2p attB4	GGGGACAACTTTGTATAGAAAAGTTGGGTGTAAGATAAGGTCAGGC	cloning
CDKG2p attB1R	GGGACTGCTTTTTTGTACAAACTTGTACAGAACTTCGGTGAGAAAGG	cloning
CDKG1 D426N Fw	GGA TTA TCC ACA GGA ATC TGA AGC CAT CT	mutating the kinase domain
CDKG1 D426N Rv	AGA TGG CTT CAG ATT CCT GTG GAT AAT CC	mutating the kinase domain
GFP qPCR Fw	TGGTCCTGCTGGAGTTCG	qPCR
GFP qPCR Rv	CTTGTACAGCTCGTCCATGC	qPCR
PP2A qPCR Fw	TAACGTGGCCAAAATGATGC	qPCR
PP2A qPCR Rv	GTTCTCCACAACCGCTTGGT	qPCR
CalS5 exon5F	GGC CTC GCA GTC TGT TAT G	Splicing
CalS5 exon7R	TTC CCT CTG GTT TCT GAC ATT	Splicing
U2AF65A_UFT	GCACAGCAGCAAATAGCTT	Splicing
U2AF65A_E12R	GGCCTGCCACTGGCTCACCATTGG	Splicing
CDKG1_RTF	GCAGTGGACATCTCAGCGTA	detection of the engogenous *CDKG1* transcript
CDKG1_mRNA_Rv	CCAGTCGACTTCCCTGTGTA	detection of the engogenous *CDKG1* transcript
