# Supplementary material for: A Functional Kinase Is Necessary for Cyclin-Dependent Kinase G1 (CDKG1) to Maintain Fertility at High Ambient Temperature in Arabidopsis
Source: Front Plant Sci. 2020 Nov 10;11:586870. doi: 10.3389/fpls.2020.586870 (PMC7683410; doi:10.3389/fpls.2020.586870)
Supplement: Supplementary file 3 [file Data_Sheet_3.pdf]

Supplementary Table 2: Anther fill and pollen viability of the different lines.

Line	Anther 1	Anther 2	Anther 3	Anther 4	Anther 5	Anther 6	
**Col-0**	+++<br>75-100%	+++<br>75-100%	+++<br>75-100%	+++<br>75-100%	+++<br>75-100%	+++<br>75-100%	anther fill<br>pollen viability
cdkg1-1	-<br>0-25%	-<br>0-25%	-<br>0-25%	-<br>25-50%	-<br>0-25%	-<br>0-25%	
***cdkg1-1* SC3**	+++<br>75-100%	+++<br>75-100%	+++<br>75-100%	+++<br>75-100%	+++<br>75-100%	+++<br>75-100%	
***cdkg1-1* SC5**	+++<br>75-100%	+++<br>75-100%	+++<br>75-100%	+++<br>75-100%	+++<br>75-100%	+++<br>75-100%	
***cdkg1-1* SC6**	+++<br>75-100%	+++<br>75-100%	+++<br>75-100%	+++<br>75-100%	+++<br>75-100%	+++<br>75-100%	
***cdkg1-1* L1**	+++<br>75-100%	+++<br>75-100%	+++<br>75-100%	+++<br>75-100%	+++<br>75-100%	+++<br>75-100%	
***cdkg1-1* L3**	+++<br>75-100%	+<br>25-50%	+++<br>75-100%	++<br>50-75%	+++<br>75-100%	+++<br>75-100%	
***cdkg1-1* L4**	+++<br>25-50%	+++<br>0-25%	++<br>0-25%	++<br>0-25%	++<br>0-25%	++<br>25-50%	
***cdkg1-1* S1**	-<br>0-25%	-<br>0-25%	-<br>0-25%	-<br>0-25%	-<br>0-25%	-<br>0-25%	
***cdkg1-1* S2**	+<br>0-25%	+<br>25-50%	+<br>25-50%	-<br>0-25%	-<br>0-25%	-<br>0-25%	
**G1p-G2-2**	-<br>0-25%	+<br>0-25%	+++<br>50-75%	++<br>25-50%	+<br>0-25%	+<br>0-25%	
**G1p-G2-3**	+++<br>50-75%	+++<br>50-75%	+++<br>0-25%	+++<br>50-75%	+<br>0-25%	+<br>0-25%	
**G2p-G2-1**	++<br>0-25%	+<br>0-25%	+++<br>75-100%	+++<br>50-75%	+++<br>50-75%	+<br>0-25%	
**G2p-G2-2**	-<br>0-25%	+<br>0-25%	-<br>0-25%	+<br>25-50%	+<br>0-25%	++<br>0-25%	
**G2p-SC12**	+++<br>50-75%	++<br>50-75%	++<br>50-75%	++<br>50-75%	+<br>50-75%	++<br>50-75%	
**G2p-SC14**	+<br>25-50%	+<br>25-50%	++<br>50-75%	+<br>25-50%	++<br>50-75%	++<br>50-75%	
**MutSC1**	-<br>0-25%	-<br>0-25%	-<br>0-25%	-<br>0-25%	-<br>0-25%	-<br>0-25%	
**MutSC2**	-<br>0-25%	-<br>0-25%	-<br>0-25%	-<br>0-25%	-<br>0-25%	-<br>0-25%	
**MutL1**	-<br>25-50%	-<br>25-50%	-<br>0-25%	-<br>25-50%	-<br>0-25%	-<br>0-25%	
**MutL5**	-<br>0-25%	-<br>0-25%	-<br>0-25%	-<br>0-25%	-<br>0-25%	-<br>0-25%	
**MutS2**	-<br>0-25%	-<br>0-25%	-<br>0-25%	-<br>0-25%	-<br>0-25%	-<br>0-25%	
**MutS3**	-<br>0-25%	-<br>0-25%	-<br>0-25%	-<br>0-25%	-<br>0-25%	-<br>0-25%	
**Col-0 SC2**	-<br>0-25%	-<br>0-25%	-<br>0-25%	-<br>0-25%	+++<br>75-100%	+++<br>75-100%	

**Col-0 SC4**	++<br>75-100%	-<br>0-25%	++<br>75-100%	+<br>25-50%	+<br>50-75%	-<br>0-25%
**Col-0 L1**	+<br>75-100%	+<br>75-100%	-<br>0-25%	++<br>50-75%	++<br>25-50%	+<br>75-100%
**Col-0 L3**	-<br>0-25%	-<br>0-25%	-<br>0-25%	-<br>50-75%	-<br>0-25%	-<br>0-25%
**Col-0 S1**	+++<br>75-100%	+++<br>75-100%	+++<br>75-100%	+++<br>75-100%	+++<br>75-100%	+++<br>75-100%
**Col-0 S4**	+++<br>75-100%	+++<br>75-100%	+++<br>75-100%	+++<br>75-100%	+++<br>75-100%	+++<br>75-100%
**Col-0MutSC1**	+++<br>75-100%	+++<br>75-100%	+++<br>75-100%	+++<br>75-100%	+++<br>75-100%	+++<br>75-100%
**Col-0MutSC2**	+++<br>75-100%	+++<br>75-100%	+++<br>75-100%	+++<br>75-100%	+++<br>75-100%	+++<br>75-100%
**Col-0MutL2**	+++<br>75-100%	+++<br>75-100%	+++<br>75-100%	+++<br>75-100%	+++<br>75-100%	+++<br>75-100%
**Col-0MutL3**	+++<br>75-100%	+++<br>75-100%	+++<br>75-100%	+++<br>50-75%	+++<br>75-100%	+++<br>75-100%
**Col-0MutS2**	+++<br>75-100%	+++<br>75-100%	+++<br>75-100%	+++<br>75-100%	+++<br>50-75%	+++<br>75-100%
**Col-0MutS3**	+++<br>75-100%	+++<br>75-100%	+++<br>75-100%	+++<br>75-100%	+++<br>75-100%	+++<br>75-100%

anther fill +++ full, ++ more than half full, + less than half full, - empty
pollen viability 0-25% , 25-50% , 50-75%, 75-100% viable pollen
